# Comparison of Root Surface Wear and Roughness Resulted from Different Ultrasonic Scalers and Polishing Devices Applied on Human Teeth: An In-Vitro Study

**DOI:** 10.3390/healthcare8010055

**Published:** 2020-03-07

**Authors:** Muhammed Bedir Mahiroglu, Erkut Kahramanoglu, Mustafa Ay, Leyla Kuru, Omer Birkan Agrali

**Affiliations:** 1 Department of Periodontology, Faculty of Dentistry, Marmara University, Istanbul 34854, Turkey; badermokresh@gmail.com (M.B.M.); lkuru@marmara.edu.tr (L.K.); 2 Department of Prosthodontics, Faculty of Dentistry, Marmara University, Istanbul 34854, Turkey; drerkut@msn.com; 3 Department of Mechanical Engineering, Faculty of Technology, Marmara University, Istanbul 34722, Turkey; muay@marmara.edu.tr

**Keywords:** dental scaling, polishing, surface roughness, ultrasonic scaling, wear

## Abstract

The aim of the present study was to compare the root surface wear and roughness, resulted from the professional dental hygiene instruments, including ultrasonic dental scalers, rubber prophy cups, and nylon bristle brushes, on the extracted human mandibular incisor teeth. Teeth (n = 80) were randomly assigned into eight groups according to the applied scaler type (Ma = Magnetostrictive, Pi = Piezoelectric), degree of power (M = Medium, F = Full), and angulation (0° and 45°). In the second stage, the specimens (n = 40) were further divided into two groups according to the applied polishing device (nylon bristle brush or rubber prophy cup). Laser scanner and contact profilometer devices were used for the surface analysis. Both ultrasonic instruments tested in our study produced rougher surfaces when full power was used at a 0° angle (*p* < 0.01). The highest wear (0.82 ± 0.07 mm^3^) and roughness values (0.30 ± 0.01 µm) were detected in the PiF0 group. Polishing performed with a rubber prophy cup resulted in almost twice the wear as well as a smoother surface when compared to polishing performed with a nylon bristle brush (*p* < 0.001). Variations in the application parameters of ultrasonic scalers and the type of polishing instrument might lead to significantly different root-surface characteristics.

## 1. Introduction

Periodontal diseases are inflammatory disorders that begin with gingivitis, progress to periodontitis, and, if left untreated, may result in irreversible destruction of tooth-supporting tissues. The microbial biofilm covering the tooth surface supports chronic inflammation and may have both local and systemic destructive potential [1]. Growing evidence regarding the two-way association between an individual’s periodontal and systemic health conditions suggests the importance of periodontal disease prevention and the need for proper treatment strategies [1].

Periodontal therapies help to restore and maintain a healthy periodontium and may also help to control related diseases. The main goal of periodontal treatment is to obtain a biologically acceptable root surface [2]. This goal can be achieved by the mechanical removal of supra/subgingival biofilm and calculus, which are the most prominent causes of periodontal disease. Ultrasonic scalers are the most common and preferred instruments for this purpose. In dentistry, there are two main ultrasonic scaler systems in which the ultrasonic vibration is obtained by either magnetostriction or piezoelectricity. However, the power settings and movement directions of these devices differ. Magnetostrictive systems operate within the range of 18 kHz to 45 kHz with an elliptical tip motion, whereas piezoelectric systems operate within the range of 25 kHz to 50 kHz with a linear motion [3]. Furthermore, the effectiveness of these tools in removing calculus from tooth surfaces varies [4]. 

Since bacterial plaque accumulates easily on rough surfaces, changes to the tooth surface may greatly affect the state of the periodontium [5,6]. Studies have shown that ultrasonic scalers result in less tissue loss but a rougher root surface when compared to hand instruments [6,7]. Conversely, no significant differences have been reported between the roughness parameters associated with hand instruments and ultrasonic piezoelectric scalers in a study analyzing the root surfaces of extracted teeth [8]. Differing results have also been obtained in studies comparing the effects of sonic and ultrasonic scalers [9,10]. Graetz et al. [9] established that the ultrasonic scaler created a smoother surface than the sonic scaler or hand instruments, whereas Ribeiro et al. [10] demonstrated that a sonic scaler with a diamond-coated tip and an ultrasonic scaler produced similar root-surface roughness, which was higher than that created with hand instruments. When we focused on the impacts of different ultrasonic instruments frequently used by professionals, we also found contradictory results. Yousefimanesh et al. [11] reported that piezoelectric scalers left smoother root surfaces than magnetostrictive scalers under the same forces. Flemmig et al. [12] also reported that a magnetostrictive device was more aggressive than a piezoelectric device on root surfaces. However, Busslinger et al. [13] reported that, after instrumentation, a piezoelectric device left a rougher root surface than a magnetostrictive device. The findings of Singh et al. [14] differed from the aforementioned studies in that magnetostrictive and piezoelectric ultrasonic tools yielded identical surface-roughness values, and their abilities for constructing a biologically harmonious surface were similar. These conflicting results highlight the need for further evaluation of this subject matter.

In current dental practice, polishing is defined as making the tooth surface smooth and glossy; it can be accomplished manually and/or by the use of engine‑driven devices, such as strips, rubber prophy cups, nylon bristle brushes, air polishers, or vector systems, in combination with numerous prophylactic pastes and powders [15,16]. Application of a prophy paste onto the tooth surface using a nylon bristle brush or rubber prophy cup as part of the mechanical periodontal treatment is the most widely used among the various polishing procedures. Polishing as the final step is believed to reduce surface roughness and remove stains and biofilm [17,18,19]. Some information is available regarding surface shifts of restorative materials after polishing procedures [20,21] and the effects of air polishers on tooth surfaces [22,23], but little attention has been paid to the comparison of rubber prophy cups and nylon bristle brushes. Chowdhary et al. [15] aimed to assess and compare the efficacy of three different polishing systems (rubber cup, bristle brush, air polisher) on enamel and on cementum surfaces using a scanning electron microscope (SEM). They reported polishing with a rubber cup resulted in superior root-surface smoothness and debris-removal than the bristle brush and air polisher. The small number of studies comparing the different polishing techniques indicates the need for further research.

In this in-vitro study, we aimed to clarify the wear and roughness effects of piezoelectric and magnetostrictive ultrasonic periodontal scalers used with different application angles and power levels followed by polishing with a nylon bristle brush or rubber prophy cup.

## 2. Materials and Methods

According to the power analysis completed using values acquired in a similar study (117.41 µm average root wear difference, 93 µm standard deviation with 80% power, significance set at *p* < 0.05) [24], a minimum of 10 teeth should be included in each group. Teeth with radicular caries, restorations extending below the cementoenamel junction (CEJ), significant anatomical variations, or external root resorption were excluded. Eighty human mandibular incisor teeth extracted in another clinic, which had hopeless prognoses and met the inclusion criteria, were used for the analysis of wear and roughness. Immediately after extraction, the teeth were cleaned in distilled water and kept in 0.1% thymol solution prior to the experimental procedures. The study was approved by the ethical committee of the Marmara University Faculty of Dentistry (No. 2018-182). For this type of study, formal consent was not required.

### 2.1. Study Groups 

This study was performed in two consecutive stages. In the first stage, eighty mandibular incisors were grouped according to the applied scaler type (**Ma** = Magnetostrictive ultrasonic scaler group; Cavitron Plus^®^, 30K FSI-SLI-1000, Slimline Insert, Dentsply International, York, PA, USA; **Pi** = Piezoelectric ultrasonic scaler group; Woodpecker^®^, UDS-A-LED, G1 Insert, Guilin Medical Instrument Co. Ltd., Guangxi, China), degree of power (**F** = Full; **M** = Medium), and angulation (**0**°; **45**°). Accordingly, the eight different groups (n = 10 teeth per group) were defined as: MaF0, MaM0, MaF45, MaM45, PiF0, PiM0, PiF45, and PiM45 (Table 1). 

In the second stage, a total of 40 specimens (five from each group showing similar wear and roughness values) were divided into two subgroups (n = 20 teeth per group) according to the applied polishing device (**Group N** = Nylon bristle brush; **Group R** = Rubber prophy cup) with polishing paste (Qartz medium grit prophylaxis paste, Dharma Research, Miami, FL, USA) (Table 1). 

### 2.2. Sample Preparation and Application Procedures

**Figure 1:**
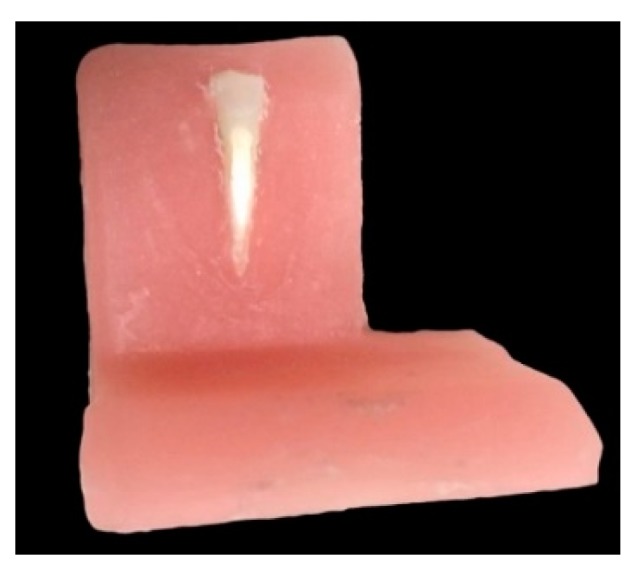
The flat platform created on a lower incisor tooth was fixed to a specially designed acrylic block.

Before the use of hygiene instruments, a flat platform was created just below the CEJ of each specimen using a grinding device (Presi Minitech 233, Presi, Eybens, France) with an abrasive paper (Reflex NAC S Type P600 Ø 250 mm, 24537, Presi, Eybens, France) for 3 s under water cooling. A specially designed setup was used to standardize instrument application. All prepared specimens were fixed to an acrylic block (Figure 1). 

**Figure 2:**
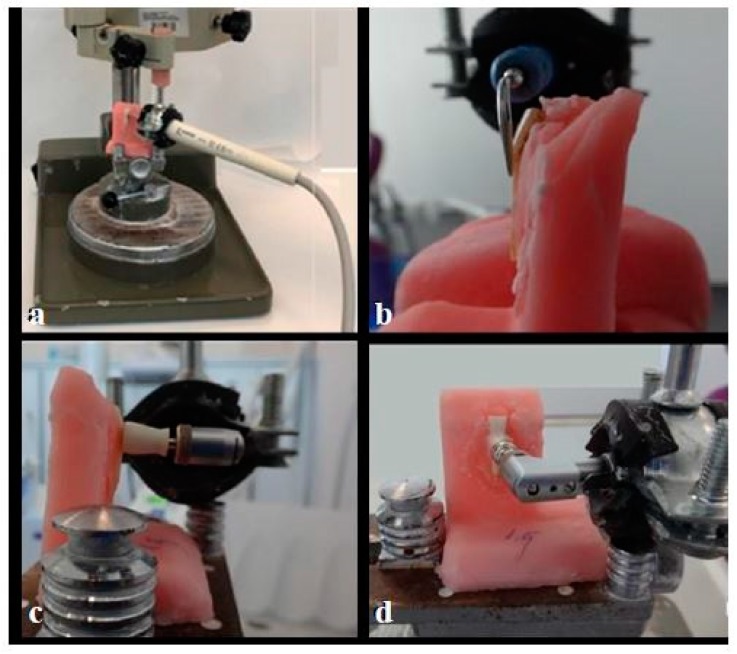
Handpieces fixed to the setup (**a**) Piezoelectric ultrasonic scaler, (**b**) Magnetostrictive ultrasonic scaler, (**c**) Rubber prophy cup, (**d**) Nylon bristle brush.

Instrumentation was standardized using a parallelometer. Handpieces were fixed tightly to the parallelometer, and the scalers were applied to each tooth surface within the respective groups for 60 s (Figure 2). Polishing devices were applied for 30 s to each specimen (Figure 2). 

### 2.3. Analysis of Wear and Roughness 

**Figure 3:**
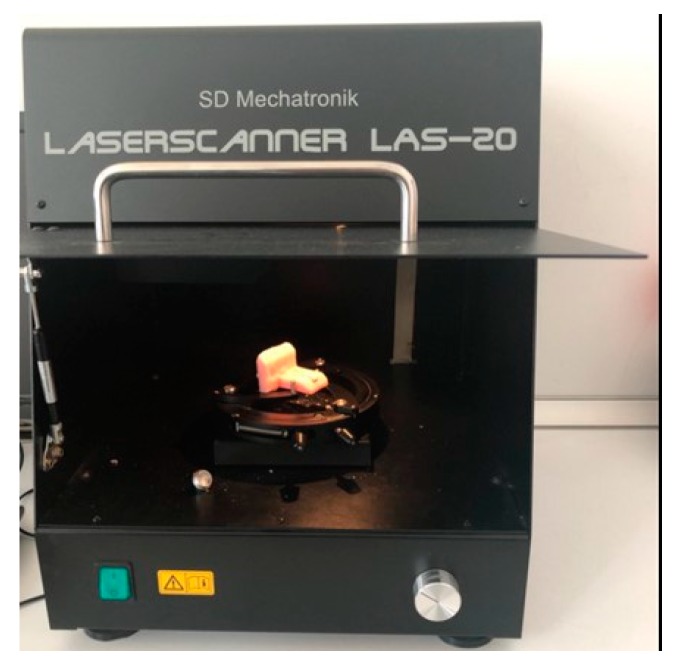
Laser scanner device

A laser scanner device (SD Mechatronic Laser Scanner LAS-20, Munich, Germany) at the Research and Development Laboratory, Department of Prosthodontics, Faculty of Dentistry, Marmara University was used to determine the amount of wear. First, the initial surface characteristics of the prepared samples were placed and fixed inside the computer-assisted laser scanner device (Figure 3).

Following identification of the start and end points marked on the samples, the 3-dimensional analysis was performed with 0.01 mm sensitivity. This was considered the baseline record.

**Figure 4:**
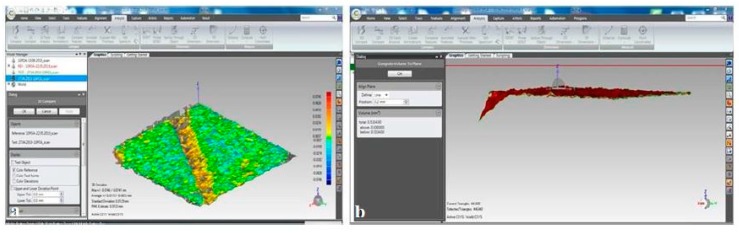
Screenshots of the software used to calculate wear values (**a**). Duplicated layers after instrumentation, (**b**). Following duplication, an imaginary line was drawn 0.2 mm away from the duplicated plane to measure the volume under the line.

After instrumentation, surface characteristics were again recorded, and the obtained data were overlapped with the baseline data to determine the amount of wear. An imaginary line was determined at 0.2 mm from the duplicated surface, and the volume under this line was calculated using a software program (Geomagic Control; Geomagic, Morrisville, NY, USA) for both before and after readings (Figure 4). The amount of wear was calculated by subtracting the revealed values.

The surface roughness (Ra: average roughness) of each sample was measured using a contact profilometer device (Perthometer M2, Mahr UK Plc, Milton Keynes, United Kingdom). Readings were verified in the marked areas of all specimens. For each reading, the needle of the device moved 1.75 mm apically within the marked area. Data regarding surface characteristics were recorded three times and averaged for each specimen before and immediately after instrumentation procedures.

### 2.4. Statistical Analysis

Data were analyzed using the Statistical Package for Social Sciences (SPSS for Windows, Release 25.0, IBM Inc., USA). Descriptive statistics are shown as the mean ± standard deviation. Normality was evaluated with the Kolmogorov–Smirnov test. The Kruskal–Wallis test was used for multiple comparisons, while the Mann–Whitney U test was used for paired comparisons. The results were interpreted with the Bonferroni correction. Statistical significance was set as *p* < 0.05.

## 3. Results

**Figure 5:**
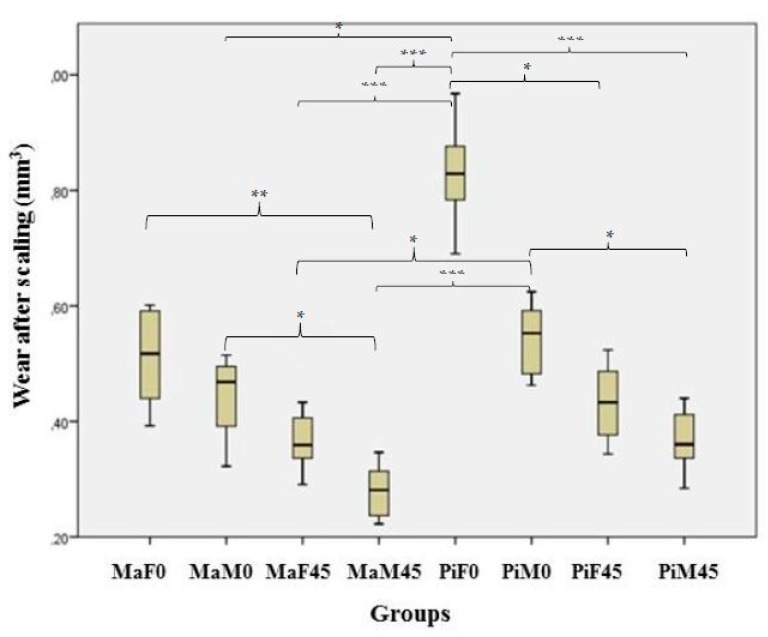
Wear values of the different scaler groups. * Mann–Whitney U posthoc Bon Ferroni Correction, *p* < 0.05; ** Mann–Whitney U posthoc Bon Ferroni Correction, *p* < 0.01; *** Mann–Whitney U posthoc Bon Ferroni Correction, *p* < 0.001.

The amount of wear on the samples was determined via laser scanning analysis with a three-dimensional drawing. The mean wear values of the different application groups are shown in Table 2 and Figure 5.

Intergroup comparison of the wear parameters revealed significant differences between the scalers (*p* < 0.01). The most pronounced wear value was detected from the piezoelectric scaler when used with full power at 0° (PiF0 group: 0.82 ± 0.07 mm^3^). The MaF0 group (magnetostrictive scaler with full power at 0°) showed almost twice the wear as the MaM45 group (magnetostrictive scaler with medium power at 45°) (*p* < 0.01) (Figure 5).

The application angle significantly affected the amount of wear in both scaler groups with medium power (magnetostrictive scaler; *p* < 0.05, piezoelectric scaler; *p* < 0.05) (Figure 5). The piezoelectric scaler applied at both power levels at 0° resulted in significantly higher wear values than the magnetostrictive scaler applied at both power levels at 45° (*p* < 0.05) (Figure 5). The piezoelectric scaler resulted in more wear when it was applied with medium power and at a working angle of 45° compared to a working angle of 0° (*p* < 0.05) (Figure 5).

A total of forty specimens were assigned to two different polishing groups. The baseline wear values were statistically similar between the groups (*p* > 0.05) (Group N = 2.57 ± 0.62 mm^3^, Group R = 2.61 ± 1.26 mm^3^). Polishing using a rubber prophy cup resulted in almost twice the wear than polishing using a nylon bristle brush (*p* < 0.001) (Table 2).

**Figure 6:**
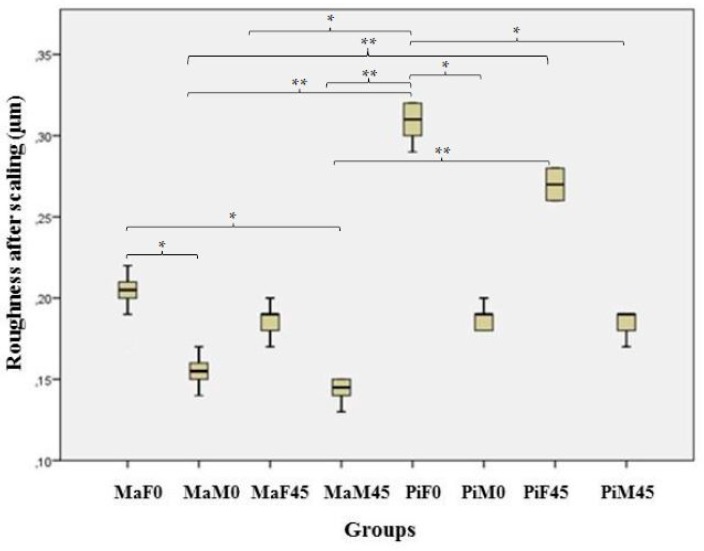
Roughness values in different scaler groups. * Mann–Whitney U posthoc Bon Ferroni Correction, *p* < 0.01; ** Mann–Whitney U posthoc Bon Ferroni Correction, *p* < 0.001.

Multiple intergroup comparison of the changes in roughness revealed significant differences (*p* < 0.001) (Table 2) (Figure 6). 

The highest roughness value was detected from the piezoelectric scaler when it was used with full power at 0° (0.30 ± 0.01 µm). The magnetostrictive scaler applied with medium power at both 0° and 45° resulted in smoother surfaces than its application with full power at 0° (*p* < 0.01) (Figure 6). The piezoelectric scaler used with full power at 0° resulted in a significantly rougher surface than both scaler types used at medium power at both working angles (*p* < 0.01) (Figure 6). The piezoelectric scaler applied with full power at 45° resulted in almost twice the roughness compared to the magnetostrictive scaler applied with medium power at both working angles (*p* < 0.01) (Figure 6). The baseline roughness values were similar between the two polishing groups (Group N = 0.40 ± 0.07 µm, Group R = 0.38 ± 0.04 µm) (*p* > 0.05). Polishing with a rubber prophy cup resulted in a significantly smoother root surface compared to polishing with a nylon bristle brush (*p* < 0.001) (Table 2). 

## 4. Discussion

In this study, we evaluated alterations on human lower incisor root surfaces that occurred immediately after the use of different types of ultrasonic scalers (applied at different angles and power settings and accompanied by the use of different polishing instruments). The most pronounced surface alterations were observed in the PiF0 group, in which the piezoelectric ultrasonic scaler was applied at full power at 0°. Moreover, the smoothest root surface was achieved after polishing with a rubber prophy cup instead of a nylon bristle brush.

Several methods, including use of an SEM [24], profilometer [25], atomic force microscope [26], digital stereomicroscope [27], three-dimensional optical laser scanner [28], and histological evaluation [8], have been proposed for analyzing tooth-surface alterations after application of periodontal hygiene instruments. No single technique has been found to deliver a complete valuation of the residual tooth surface, and each method suffers its own limits [29]. In the present study, the amount of wear was identified using a three-dimensional optical laser scanner, while the surface roughness value was measured using a contact profilometer device. To our knowledge, we are the first to analyze tooth-surface change after the use of hygiene instruments using volumetric subtraction measurement and software to calculate the wear value revealed by the optical laser scanner.

The mean Ra value, which represents the arithmetic average of complete values of the contour deviations from the mean plane contained in the sampling region, is measured in µm; we chose to use it in our study because it is the most commonly used parameter for surface roughness. However, the definition of the Ra value depends on the area to be scanned. Furthermore, individual errors in the relevant area to be scanned may provide different measurement values [30]. Kocher et al. [30] aimed to define the circumstances and need for the three-dimensional roughness measurement of tooth-root surfaces using a laser profilometer. They reported that revealed values were intensely reliant on the measurement conditions, and, therefore, the outcome of one study could not be directly compared to another. In light of this, although the application angle significantly affected the amount of wear in both scaler groups at medium power in our study (MaM0 = 0.44 ± 0.06 mm^3^, MaM45 = 0.28 ± 0.04 mm^3^, PiM0 = 0.54 ± 0.05 mm^3^, PiM45 = 0.36 ± 0.05 mm^3^), it was deemed appropriate to discuss the influences of the ultrasonic scalers applied at the same angle; the dimension of the contact area formed between the tip of different types of ultrasonic scalers and the tooth differed [24]. Thus, the effects of scaler type and different power settings on the surface would be better presented. Admittedly, the contact surface area of the tip applied at 45° differed from that applied at 0°. Regarding roughness, both ultrasonic instruments tested in our study produced rougher surfaces when the power setting was changed from medium to full level at 0°. The highest roughness value was detected in the PiF0 group (MaM0 = 0.15 ± 0 µm, MaF0 = 0.20 ± 0.01 µm; PiM0 = 0.18 ± 0 µm, PiF0 = 0.30 ± 0.01 µm). Consistent with this finding, Casarin et al. [31] identified a positive correlation between power settings and roughness values for ultrasonic scalers. Conversely, Kumar et al. [28] did not establish any direct correlation between the surface roughness and power settings of ultrasonic scalers. These inconsistent findings might be due to differences in the individual application methods of each study. When the obtained roughness values were evaluated based on the comparison of scaler type, the piezoelectric ultrasonic scaler applied with full power at 0° resulted in almost twice the roughness when compared to the magnetostrictive ultrasonic scaler applied with medium power at 0° (PiF0 = 0.30 ± 0.01 µm and MaM0 = 0.15 ± 0 µm, respectively). Similarly, at 45°, the magnetostrictive ultrasonic scaler applied with medium power resulted in a surface almost twice as smooth as the piezoelectric ultrasonic scaler applied with full power (MaM45 = 0.14 ± 0 µm and PiF45 = 0.27 ± 0 µm, respectively). These findings were in line with the studies suggesting different surface roughness results are due to differences in adjusted lateral force, tip angulation, and power settings [11,12,24,32]. On the other hand, in our study, the ultrasonic scaler type did not significantly influence surface roughness. This result was inconsistent with the limited number of comprehensive studies in the literature, particularly those comparing the surface roughness effects of the magnetostrictive and piezoelectric ultrasonic scalers suggesting differences are due to varying impacts [11,13]. Busslinger et al. [13] reported that the piezoelectric ultrasonic scaler was more efficient than the magnetostrictive ultrasonic scaler in eliminating calculus but left the instrumented surface rougher. On the contrary, Yousefimanesh et al. [11] demonstrated that, under the same forces, a piezoelectric scaler created smoother surfaces than a magnetostrictive device. These inconsistent results, which differed from those of our study, might be due to variances in application durations and forces. In the present study, although changes in the application angle might have resulted in different surface contact areas [24], tip angulation did not give rise to a significant effect on surface roughness in groups of the same ultrasonic scaler type and power settings. Oliviera et al. [24] evaluated the roughness and wear effects of ultrasonic scalers applied at different angles, such as 30°, 40°, 60°, and 90°, and reported that an ultrasonic scaler used at 90° left a smoother surface than at other working angles. In addition, they stated that an increase in the working angle resulted in a rougher surface due to the increased force against the root surface. The inconsistency with our study might be explained due to the prevention of individual errors; this was accomplished in our study using a special set-up to maintain constant contact with the tooth surface.

In the present study, the only statistically significant difference in wear effect was found between the magnetostrictive scaler applied at medium power and the piezoelectric scaler applied at full power, both at 0° (*p* = 0.033). Within these settings, the piezoelectric ultrasonic device removed almost two times the amount of tooth substance from the root surface compared to the magnetostrictive ultrasonic device (MaM0 = 0.44 ± 0.06 mm^3^, PiF0 = 0.82 ± 0.07 mm^3^). Following the use of power-driven ultrasonic devices on the root surface, a number of variables, such as generator power, applied force, angulation, time extent, tip form, and tip motion, make root-surface comparisons practically impossible [14,33]. Similar to our roughness findings, we found that changing the ultrasonic scaler type did not exert any significant influence on root-surface wear at the same power settings and application angles (*p* > 0.05). Although there are studies in the literature investigating the wear effects of ultrasonic instruments on root surfaces under different operating conditions [12,25,32,34,35], until now, no study has specifically compared the wear impacts of magnetostrictive and ultrasonic scalers under the same circumstances.

Determination of surface changes after polishing with a nylon bristle brush and rubber prophy cup might be considered the second-most important part of the current study. We compared 20 samples per group, showing similar baseline wear and roughness properties. The smoothest surface and the most notable wear effect were achieved in Group R. This was the expected result, which could be explained by the decrease in deviations from the mean plane, resulting in a volumetric increase calculated by the software. In addition, our polishing results were in line with a study by Chowdhary et al., comparing the effects of similar polishing instruments on the root surface [15].

This study presented certain limitations, such as the absence of application angles other than 0° and 45° and time settings other than 60 s. There was also a lack of lateral force executions. However, it should be kept in mind that the lateral force implementation is not easily replicated or standardized [36]. Nevertheless, within these limits, our findings provided information on certain comparisons, including root-surface alterations after the use of widely preferred conventional dental hygiene instruments. Revealed results might guide the professionals to choose the accurate hygiene instruments and working parameters, leading to minimal root surface alterations. Accordingly, the use of a piezoelectric dental scaler at full power might cause significant wear and roughness effects. It might be considered that the rubber prophy cup preference might be a better option in the polishing process after using this professional hygiene tool in such a condition compared to the nylon bristle brush. Further studies are needed to investigate the effects of different dental hygiene instruments on the surface of the human teeth under similar conditions. In addition, making the measurement criteria clear and reproducible for the standardization of future studies in which root surface alterations will be examined is of great importance in order to establish healthy comparisons.

## 5. Conclusions

We conclude that magnetostrictive and piezoelectric ultrasonic scalers result in similar wear and roughness effects on root surfaces when used under the same conditions. Changes in the application parameters of ultrasonic scalers may lead to significant differences in their impacts on root surfaces. It has been observed that, under the same time period, the change in the power setting parameter has a more significant effect than the change in the angle, especially on roughness results. Attention should be paid to the negative consequences after the use of a piezoelectric ultrasonic scaler at a zero degree angle on the root surface. Furthermore, a rubber prophy cup may be a more effective polishing tool than a nylon bristle brush since it creates a smoother root-surface texture.

## Figures and Tables

**Table 1:** Study groups.

STAGE 1 (80 Teeth)								
MaF0 (n = 10)	MaM0 (n = 10)	MaF45 (n = 10)	MaM45 (n = 10)	PiF0 (n = 10)		PiM0 (n = 10)	PiF45 (n = 10)	PiM45 (n = 10)
Magnetostrictive Scaler Full Power 0 Degrees	Magnetostrictive Scaler Medium Power 0 Degrees	Magnetostrictive Scaler Full Power 45 Degrees	Magnetostrictive Scaler Medium Power 45 Degrees	Piezoelectric Scaler Full Power 0 Degrees		Piezoelectric Scaler Medium Power 0 Degrees	Piezoelectric Scaler Full Power 45 Degrees	Piezoelectric Scaler Medium Power 45 Degrees
**STAGE 2** **(40 Teeth)**								
**Group N** **(n = 20)**					**Group R** **(n = 20)**			
Polishing with a nylon bristle brush					Polishing with a rubber prophy cup			

**Table 2:** Wear and roughness of different ultrasonic scalers and polishing groups.

	Scaling Groups									
	MaF0 (n = 10)	MaM0 (n = 10)	MaF45 (n = 10)	MaM45 (n = 10)	PiF0 (n = 10)		PiM0 (n = 10)	PiF45 (n = 10)	PiM45 (n = 10)	*p* *
Wear Mean ± SD (mm^3^)	0.50 ± 0.08	0.44 ± 0.06	0.37 ± 0.07	0.28 ± 0.04	0.82 ± 0.07		0.54 ± 0.05	0.43 ± 0.06	0.36 ± 0.05	0
Roughness Mean ± SD (µm)	0.20 ± 0.01	0.15 ± 0	0.18 ± 0	0.14 ± 0	0.30 ± 0.01		0.18 ± 0	0.27 ± 0	0.18 ± 0	0
	**Polishing groups**									
	**Group N** **(n = 20)**					**Group R** **(n = 20)**				***p*^#^**
Wear Mean ± SD (mm^3^)	0.04 ± 0.01					0.10 ± 0.02				0
Roughness Mean ± SD (µm)	−0.08 ± 0					−0.11 ± 0.01				0

* Kruskal–Wallis Test, *p* < 0.001 ^#^ Mann–Whitney U Test, *p* < 0.001.
